# Short-term effects of a 12-week Zumba program on thyroid and cardiometabolic markers in overweight/obese postmenopausal women: A randomized pilot study

**DOI:** 10.1016/j.clinsp.2026.100880

**Published:** 2026-02-19

**Authors:** Hanen Saadouni, Okba Selmi, Nejmeddine Ouerghi, Hamza Marzouki, Soukaina Hattabi, Lamia Attig, Katja Weiss, Thomas Rosemann, Beat Knechtle, Anissa Bouassida

**Affiliations:** a Research Unit “Sport Sciences, Health and Movement” (UR22JS01) High Institute of Sport and Physical Education of Kef, University of Jendouba, 7100 Kef, Tunisia; b University of Tunis El Manar, Faculty of Medicine of Tunis, Rabta Hospital, LR99ES11, 1007 Tunis, Tunisia; c University of Gafsa, High Institute of Sport and Physical Education of Gafsa, 2100 Gafsa, Tunisia; d High Institute of Sport and Physical Education of Kef, University of Jendouba, Tunisia; e CI-ISCE, Higher Institute of Educational Sciences of the Douro, 4560-547 Penafiel, Portugal; f Institute of Primary Care, University Hospital of Zurich, Zurich, Switzerland

**Keywords:** Cardiovascular health, Thyroid hormones, Menopause lipids, Obese, Zumba fitness

## Abstract

**Objective:**

This pilot study aimed to investigate the effects of a 12-week Zumba training program on Thyroid-Stimulating Hormone (TSH), Free Thyroxine (FT4), lipid profile, and anthropometric parameters in overweight and obese postmenopausal women.

**Methods:**

Twenty-two women (aged 44‒60 years) were randomly assigned to a Zumba training group (ZG; *n* = 11) or a control group (CG; *n* = 11). The ZG participated in 60-min Zumba sessions three times per week for 12-weeks, while the CG maintained their usual lifestyle. Before and after the intervention, participants underwent assessments of body composition, blood pressure, and circulating levels of TSH, FT4, Total Cholesterol (TC), and Triglycerides (TG).

**Results:**

Following the intervention, the ZG showed significant reductions in TSH (*p* = 0.001; Cohen’s d (*d)* = 0.90), TG levels (*p* = 0.043; *d* = 0.75), body fat percentage, body mass index, waist and hip circumference, and both systolic and diastolic blood pressure (all *p* < 0.05). No significant changes were observed for FT4 and TC levels. No notable changes were recorded in the CG.

**Conclusions:**

The 12-week Zumba program can serve as a feasible and enjoyable strategy for promoting physical activity in overweight and obese postmenopausal women, leading to improvements in thyroid function, TG levels, body composition, and blood pressure. These preliminary findings support the potential of Zumba for enhancing cardiometabolic health during menopause, though larger, long-term trials are needed to confirm these effects and investigate their durability.

## Highlights

• Zumba lowers TSH and triglycerides in postmenopausal obese women.
• Zumba improves body composition and blood pressure after menopause.
• Zumba is a non-drug strategy to boost postmenopausal women's health.
• Dance-based training helps regulate thyroid and lipid levels postmenopause.

## Introduction

Menopause is a physiological and biological process affecting women aged 45- to 55-years, characterized by the cessation of menstruation due to declining reproductive hormones, marking the end of fertility.^1^ These hormonal changes have significant metabolic consequences, including an increased risk of obesity, hypertension, hypercholesterolemia, type 2 diabetes, and cardiovascular disease.^2^, ^3^, ^4^ Importantly, menopause also affects thyroid function, which is crucial for regulating metabolism.^5^

The thyroid gland, in conjunction with the pituitary gland, serves as the primary regulator of weight and metabolic processes.^6^ Thyroid hormones regulate vital bodily functions, including respiration, heart rate, nervous system function, muscle strength, menstrual cycle, temperature regulation, and cholesterol levels^7^ During menopause, the decrease in estrogen and progesterone alters these functions and affects thyroid hormone production, increasing the risk of thyroid dysfunction among postmenopausal women.^8^^,^^9^ This often manifests as subclinical hypothyroidism, characterized by normal Free Thyroxine (FT4) but elevated Thyroid-Stimulating Hormone (TSH).^10^^,^^11^ Even mild thyroid insufficiency can lead to clinical symptoms such as depression, memory loss, cognitive impairment, and neuromuscular disorders.^12^

While hormonal treatments are available for managing menopausal symptoms, regular physical activity remains one of the most effective non-hormonal interventions for menopausal women. However, identifying appropriate exercise modalities that address both menopausal and obesity-related concerns remains challenging. Zumba fitness has emerged as a popular physical activity among women, combining dance and fitness movements with Latin and international rhythms.^13^^,^^14^ This program incorporates key elements of physical fitness, such as strength, endurance, flexibility, and balance in an engaging format.^15^ Studies have shown that music-based exercise, like Zumba, enhances psychological well-being, reducing anxiety and depression.^16^^,^^17^ The comprehensive nature of Zumba, incorporating aerobic, fast, and balanced movements, makes it a holistic mind-body exercise that addresses various physical, psychosocial, and behavioral aspects of health.^18^^,^^19^ Scientific evidence has demonstrated numerous positive outcomes of Zumba interventions in healthy women, including improvements in body composition, cardiovascular fitness, physical performance, and psychological well-being.^20^, ^21^, ^22^, ^23^ Based on previous findings, aerobic dance-based interventions such as Zumba have shown greater influence on Triglyceride (TG) levels than on Total Cholesterol (TC), particularly in overweight or sedentary individuals.^22^^,^^24^ Despite the crucial role thyroid hormones play in the health of obese postmenopausal women, scant research has explored the impact of physical exercise on these hormones in this demographic.^25^^,^^26^ Notably, there is a lack of studies investigating how Zumba exercise interventions influence thyroid hormone and thyroid-stimulating hormone concentrations in obese menopausal women.

The present pilot study aims to address this gap by examining the effects of a 12-week Zumba training program on thyroid hormones, body composition, and TC and TG levels in overweight and obese postmenopausal women. The authors hypothesize that this intervention will improve not only body composition and lipids but also thyroid function in this population. Specifically, the authors expected that Zumba training would primarily reduce TG levels, with potential but less predictable effects on TC.

## Methods

### Participants

Prior to participant recruitment, a sample size estimation was performed using G*Power software (version 3.1.9.4; University of Kiel, Kiel, Germany). Based on the study design (repeated-measures ANOVA with within-between interaction) and a large effect size (*f* = 0.40) derived from published data,^27^^,^^28^ the analysis determined that a minimum of eight participants per group was needed (actual power = 80.70 %) to detect significant differences, assuming a Type I error rate of 0.05 and a Type II error rate of 0.20 (statistical power = 80 %). Thus, thirty overweight or obese postmenopausal women, aged 44–60 years, voluntarily participated in the study. They were randomized into two groups: Zumba group (ZG; performing a Zumba training, *n* = 15) and control group (CG; maintaining their usual activities for 12-weeks, *n* = 15). Of the 30 selected participants, eight women did not complete the experiment for personal reasons (four from the ZG and four from the CG). Consequently, 22 participants completed the experimental protocol: 11 in ZG (age: 52.6 ± 4.31 years; height: 1.61 ± 0.044 m) and 11 in CG (age: 55.7 ± 4.33 years; height: 1.60 ± 0.048 m) (Fig. 1).

**Fig.. 1:**
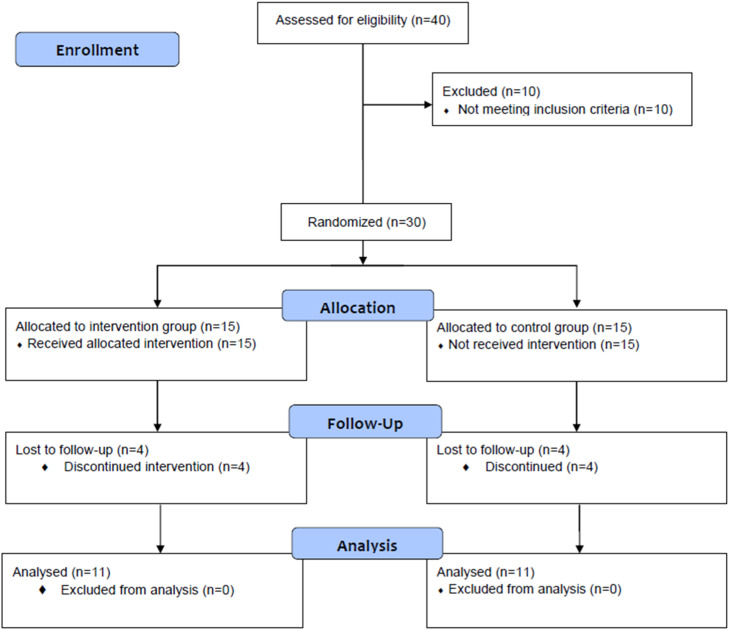
Consort flow diagram.

The inclusion criteria were as follows: ages 44- to 60-years; body mass index ≥ 25 kg/m^2^; a self-reported amenorrhea for at least 3-years, based on clinical history; and a stable heart rate. The exclusion criteria were as follows: therapy for obesity, chronic illnesses, endocrine disorders, diabetes mellitus, or contraindications to training. All participants underwent a comprehensive medical evaluation prior to inclusion, including clinical history and resting heart rate assessment. Women with comorbidities such as cardiovascular, metabolic, thyroid, or inflammatory diseases, as well as those receiving medications that could influence metabolic or hormonal parameters, were excluded. The local research ethics committee of the High Institute of Sport and Physical Education of Kef (ISSEPK), Tunisia (Approval Number: ISSEPK/12-2023), approved the study, adhering to the ethical guidelines of the Helsinki Declaration for human research. Throughout the intervention period, participants were instructed to maintain their usual dietary habits, and no specific monitoring of dietary intake was carried out.

### Design

The study was conducted from June to September 2023, and it was designed to evaluate the efficacy of a 12-week Zumba training program on anthropometric and biochemical markers in postmenopausal women. Anthropometric measurements (body height, body mass, Body Mass Index (BMI), skinfold thickness, waist, hip, and chest circumferences), blood pressure, thyroid hormones, total cholesterol, and triglycerides were evaluated before and after the 12-week interventions. One week before starting the experimentation, all participants completed three separate familiarization sessions with the experimental procedures (∼48 h) to ensure their technical proficiency in performing testing and training procedures, and with the scale of Perceived Exertion (RPE).^29^

### Anthropometric measures

All anthropometric measurements were taken by one trained researcher using the same equipment to minimize variability. Measurements of women’s height and body mass were conducted with the participants barefoot and wearing only light clothing. Height was recorded in centimeters with an accuracy of 0.1 cm using a Harpenden stadiometer. During the height measurement, the women stood in an upright position, with their heels together, arms extended, and heads positioned parallel to the floor. Body mass was recorded in kilograms (measured to the nearest 0.1 kg) using an electronic scale (Tanita, Model TBF-410 GS, Tokyo, Japan). During the body mass measurement, participants stood upright at the center of the scale platform with their arms extended. BMI was calculated as body mass divided by height squared (kg/m^2^).

Skinfolds (triceps, biceps, subscapular, supra-iliac) were measured by a single trained assessor using a calibrated Harpenden-type caliper (Holtain Instruments, Crosswell, Pembrokeshire, UK) following a standardized protocol. Each site was assessed three times in rotation**;** the median of three was used for analysis. Body density was estimated according to the equations of Durnin & Womersley.^30^ Body fat percentage was calculated using Siri’s equation.^31^ These procedures are recommended to reduce technical error and maximize intra-observer reliability in field settings. Only the right side of the body was used for unilateral skinfold thickness measurements to ensure consistency in the measurement method. Waist, hip, and chest circumferences were measured. Waist circumference was measured at the smallest circumference between the rib cage and the iliac crest, with the subject standing. Hip circumference is taken at the widest area of the hips at the greatest protuberance of the buttocks.

### Blood pressure

Systolic and diastolic measurements were obtained using an arm tensiometer (Exacto KD 591; Biosynex, Strasbourg, France). The cuff was securely attached to each participant’s left forearm while they were seated in a chair with a backrest. The arm was parallel to the table and at heart level. After a 15-min rest period, measurements were taken while the subjects remained seated.^32^

### Zumba training

The experimental group participated in a 12-week Zumba training program conducted from June to September. Sessions were scheduled three times a week on Mondays, Wednesdays, and Fridays from 5 pm to 6 pm and were led by a certified Zumba instructor whose qualifications included specific certification in Zumba and extensive experience in fitness instruction. Each session commenced with a 15-min warm-up comprising rhythmic movements set to music, including step touch, double step touch, side to side, leg curl, double leg curl, knee up, double knee up, V step, squat, and hop, with music ranging from 120 to 135 beats per minute. The main session focused on choreographed routines led by the instructor, incorporating fundamental Zumba steps such as step, salsa, mambo, tango, turns, grapevine steps, and cha-cha-cha.^23^The choreographies primarily utilized routines from songs numbered 48, 50, and Love ZIN75 on DVD, set to music ranging from 140 to 180 beats per minute. Each dance segment lasted between 3- and 5-min, interspersed with rest intervals of 15–30 s.^23^ This variety engaged different muscle groups, including the thighs, buttocks, abdomen, and arms throughout the sessions. The final part of each session, lasting 15-min, involved light dance movements to music with a tempo of 100 bpm, aimed at gradually lowering heart rates and inducing mental and psychological relaxation through soothing music. The initial weeks focused on mastering basic movements without rotations or jumps, with session durations starting at 25-minutes and progressively increasing to 35-minutes over the final four weeks as choreographic complexity increased. To standardize internal load, instructors coached participants to maintain Borg RPE 12‒14 (“somewhat hard”) for most choreography blocks and 15‒16 (“hard”) during brief peaks, ranges that correspond to the first and second ventilatory thresholds in adult aerobic exercise.^33^, ^34^, ^35^ This approach has been validated as a proxy for HR and VO_2_ in older and overweight/obese populations performing rhythmic aerobic activity.^36^, ^37^, ^38^, ^39^ and was chosen to ensure a moderate-to-vigorous dose in a pragmatic, group-based setting.

### Blood sampling and methods of analysis

Fasting venous blood samples were collected from an antecubital vein in the morning, before and after completing the 12-week exercise program. Samples were centrifuged and frozen at −80 °C until analysis (within 3-months). Concentrations of TSH and FT4 were measured using the Access Automated Immunoassay System (Access2, USA). TC and TG samples were taken in heparin-containing tubes. They were measured in plasma using a biochemistry automaton (Beckman Coulter AU480) and enzymatic colorimetric methods, utilizing the respective reagent kits (Beckman Coulter) Fig. 1.

### Statistical analysis

Statistical analyses were performed using the SPSS software version 22.0 (SPSS, Inc., Chicago, IL, USA). Data were expressed as means ± Standard Deviations (SD). The Shapiro-Wilk test assessed the normality of the data, while Levene’s test was used to evaluate the homogeneity of variances. A two-way repeated measure ANOVA (2-times × 2-conditions) was applied to all outcome variables. Effect sizes for ANOVA were reported as partial eta-squared (η²_p_) and interpreted as follows: small (0.01 < η²*_p_* < 0.06), medium (0.06 ≤ η²*_p_* < 0.14), and large (η²*_p_* ≥ 0.14).^40^ In cases of significant interaction effects, Bonferroni post-hoc tests were performed on all variables. To compare percentage changes (∆) between groups, independent-samples *t*-tests were conducted. Cohen’s d (*d*) was calculated to quantify the magnitude of the differences within and between groups, and was classified according to Cohen as trivial (< 0.20), small (0.20 ≤ *d* < 0.50), medium (0.50 ≤ *d* < 0.80), and large (≥ 0.80) magnitudes.^40^ The level of statistical significance was set at *p* ≤ 0.05.

## Results

Normality of the data and homogeneity of the variances were confirmed (all *p* > 0.05).

Before training, there were no significant differences between the two groups (ZG and CG) for any of the measured variables (Table 1).

**Table 1:** Anthropometric and biochemical parameters before and after Zumba training program in overweight/obese postmenopausal women.

	Control group (*n* = 11)			Zumba group (*n* = 11)			Interaction (time × group)		
	Before	After	*∆* (%)	Before	After	*∆* (%)	*F*	*p*	*η²_p_*
**Age (years)**	55.7 ± 4.33		‒	52.6 ± 4.31		‒	‒	‒	‒
**Height (m)**	1.60 ± 0.048		‒	1.61 ± 0.044		‒	‒	‒	‒
**Body mass (kg)**	80.0 ± 16.1	80.5 ± 17.2	0.45	83.0 ± 13.2	81.1 ± 12.4^a^	−2.20	8.95	0.007	0.309
**Body mass index (kg/m^2^)**	30.9 ± 5.40	31.2 ± 5.9	0.45	31.8 ± 5.00	31.1 ± 4.83^a^	−2.21	9.30	0.006	0.317
**Fat mass (kg)**	30.2 ± 7.87	30.6 ± 9.13	0.56	31.9 ± 8.55	29.6 ± 8.53^a^	−7.26	9.14	0.007	0.314
**Lean mass (kg)**	49.8 ± 8.66	50.0 ± 8.46	0.35	51.1 ± 5.72	51.5 ± 4.95	0.82	0.11	0.744	0.005
**Body fat (%)**	37.1 ± 2.88	37.5 ± 3.32	0.07	37.9 ± 4.77	35.9 ± 5.38^a^	−5.21	6.39	0.020	0.242
**Waist circumference (cm)**	99.5 ± 13.3	99.3 ± 13.83	−0.22	93.8 ± 9.06	91.5 ± 10.0^b^	−2.60	5.33	0.032	0.211
**Hip circumference (cm)**	113.9 ± 10.2	114.1 ± 10.5	0.15	111.5 ± 10.5	110.3 ± 10.2^a^	−1.12	4.87	0.039	0.196
**Chest circumference (cm)**	99.9 ± 11.80	100.2 ± 11.84	0.32	102.9 ± 9.22	101.1 ± 9.10^b^	−1.76	25.40	<0.0001	0.559
**Systolic blood pressure (mm Hg)**	132.1 ± 4.84	132.6 ± 6.57	0.40	134.4 ± 6.60	125.7 ± 7.52^b^	−6.34	15.12	0.001	0.431
**Diastolic blood pressure (mm Hg)**	82.0 ± 5.38	81.7 ± 5.12	−0.30	83.7 ± 6.63	76.0 ± 6.67^b^	−8.89	10.72	0.004	0.349
**Total Cholesterol (mg/dL)**	191.0 ± 37.7	197.0 ± 31.8	4.30	187.0 ± 29.7	182.7 ± 32.4	−1.88	1.33	0.263	0.062
**Triglycerides (mg/dL)**	116.3 ± 27.3	144.4 ± 57.3	28.93	124.1 ± 59.3	90.5 ± 29.9^a,d^	−19.52	7.55	0.012	0.247
**Thyroid stimulating hormone (mUI/L)**	2.53 ± 0.95	2.90 ± 1.32	14.11	3.51 ± 1.37	2.4 ± 1.22^c^	−31.49	16.57	0.001	0.453
**Free thyroxine (pmoL/L)**	9.99 ± 0.92	9.73 ± 0.44	−1.92	9.94 ± 1.53	10.0 ± 1.53	2.14	0.57	0.461	0.028

Values are expressed as mean ± SD. ∆, Percentage change.

a *p* < 0.05, b *p* < 0.01, c *p* < 0.001: before vs. after; d *p* < 0.01: Zumba group vs. control group.

Statistical analysis showed a significant time × group interaction (*p* < 0.05) for body composition parameters and blood pressure (Table 1). Compared to the baseline values, body mass (*p* = 0.019, *d* = 0.16), BMI (*p* = 0.015, *d* = 0.15), fat mass (*p* = 0.012, *d* = 0.28), body fat percentage (*p* = 0.017, *d* = 0.40), waist circumference (*p* = 0.008, *d* = 0.25), hip circumference (*p* = 0.026, *d* = 0.20), chest circumference (*p* = 0.001; *d* = 0.23), systolic (*p* = 0.002, *d* = 1.29), and diastolic (*p* = 0.007, *d* = 1.21) blood pressure improved in ZG (Table 1).

For circulating levels of TSH and triglycerides, a significant time × group interaction was found (*p* < 0.05). However, no significant interaction (*p* > 0.05) was observed for the circulating levels of total cholesterol and FT4. Compared to the baseline values, the circulating levels of TSH and triglycerides significantly decreased (*p* = 0.001; *d* = 0.90 and *p* = 0.043; *d* = 0.75, respectively) after a 12-week Zumba training program. No significant variations were observed for any variables in the control group. In the between-group comparison, statistical analysis indicated a lower level of TG in ZG compared to CG after the intervention (*p* = 0.01; *d* = 1.24) (Table 1). Fig. 2 displays the changes in biochemical variables induced by the Zumba training program.

**Fig.. 2:**
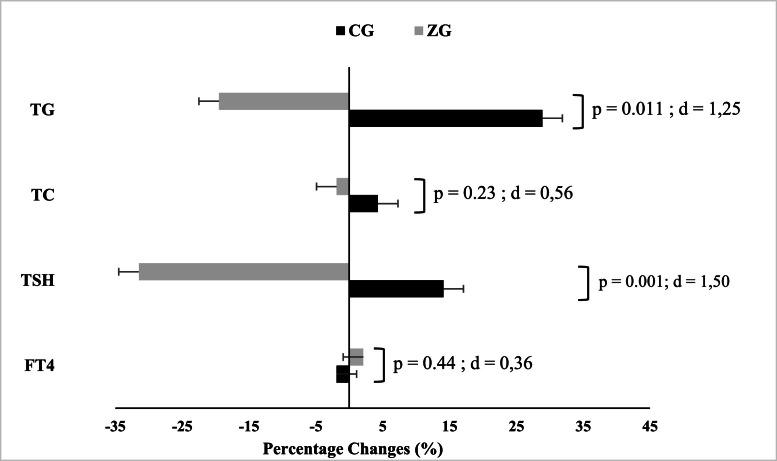
Mean percentage changes in Total Cholesterol (TC), Triglycerides (TG), Thyroid-Stimulating Hormone (TSH), and Free Thyroxine (FT4) following a 12-week intervention program in Zumba training (ZG) and Control (CG) groups. d, Cohen’s d. Negative values indicate a reduction relative to baseline. The statistical significance level was set at *p* ≤ 0.05.

## Discussion

To the best of the author's knowledge, no previous research has investigated the effects of a 12-week Zumba training program on thyroid hormones in overweight/obese postmenopausal women. The main findings revealed that the program decreased circulating TSH levels but did not significantly affect FT4 levels. Concurrently, certain health markers (body composition, TG levels, and blood pressure) significantly improved following this intervention in postmenopausal women.

### Effect of the Zumba intervention on thyroid hormone

To date, no studies have assessed the effects of Zumba training on TSH and FT4 levels in overweight or obese postmenopausal women. The present study showed that 12-weeks of Zumba training significantly reduced circulating TSH levels but had no significant effect on FT4 levels. These findings are consistent with other interventional training studies showing significant decreases in TSH levels after 12-weeks of aerobic training,^41^ or high-intensity interval training,^25^ in obese boys and adolescent girls, respectively. In the same context, 16-weeks of resistance training showed a significant decrease in serum TSH concentration levels in school children.^42^ On the other hand, other studies showed no significant change in TSH and thyroxine (T4) levels after 12-weeks of aerobic exercise with music in obese women.^43^ A study by Barari^44^ reported that 8-weeks of endurance training had no significant effect on TSH, Triiodothyronine (T3), and T4 levels in obese men. However, another study showed that 12-weeks of endurance training increased TSH and T4 levels in male athletes.^45^ Differences in subject characteristics and training methods may explain the variations between these studies.

The precise mechanisms underlying the observed reduction in TSH following Zumba training remain unclear and were not directly investigated in this study, as the authors did not measure key regulators such as leptin, TRH, T3, or tissue-level thyroid hormone activity. Therefore, these findings demonstrate an association rather than establishing causation. The concurrent improvements in body composition and TSH levels suggest a potential link between fat mass reduction and modulation of the Hypothalamic-Pituitary-Thyroid (HPT) axis, as previously suggested in the literature.^46^, ^47^, ^48^ It is plausible that exercise-induced metabolic improvements could influence central regulatory pathways, but this remains speculative. Future studies incorporating measurements of leptin, full thyroid profiles (including T3), and direct assessments of HPT axis activity are necessary to elucidate the causal mechanisms responsible for the exercise-induced modulation of thyroid hormones observed here.

An important null finding is that FT4 did not change despite a modest reduction in TSH, with both remaining within reference ranges. In euthyroid adults, FT4 is tightly buffered; short-term exercise adaptations may manifest first as altered pituitary drive or peripheral hormone economy without detectable shifts in circulating FT4.^25^^,^^43^^,^^49^^,^^50^ This stability is also compatible with the relatively long half-life and homeostatic control of FT4 over a 12-week period. Accordingly, these findings are more consistent with physiological modulation of the HPT axis tone than with increased thyroid hormone secretion. The concurrent improvements in TG, body composition, and blood pressure may therefore arise primarily from exercise-induced cardiometabolic adaptations independent of changes in FT4. Given the small sample and short duration, the study was not powered to detect small FT4 changes; longer, adequately powered trials with comprehensive thyroid panels (including T3 and reverse T3) and mechanistic endpoints are warranted.

Emerging research further underscores the relevance of thyroid function in the context of gynecologic endocrinology. Melatonin, which may be stimulated by physical activity like Zumba, plays a protective role in the thyroid gland, notably by attenuating oxidative stress and potentially modulating autoimmune processes.^51^ Its positive effects on sleep quality, estradiol levels, and BMI in postmenopausal women also support an indirect regulatory role in thyroid function.^51^ Additionally, menopause itself is associated with alterations in thyroid hormone dynamics, independent of overt thyroid disease, which may result in elevated TSH levels and reduced thyroid responsiveness to stimulation.^52^ Furthermore, thyroid dysfunctions are increasingly recognized as interlinked with reproductive health and inflammatory states. For example, even subtle thyrocyte dysfunction may coincide with inflammatory hematologic disturbances in benign thyroid nodules, suggesting that systemic low-grade inflammation could be a modifiable target of exercise interventions.^53^ This aligns with the broader view that thyroid health is a critical factor in postmenopausal homeostasis and reproductive-endocrine interactions.^54^

All thyroid parameters in this study remained within the euthyroid range, indicating minor yet potentially beneficial physiological adaptations. Given the age-related decline in FT4 production and thyroid sensitivity to TSH, the observed decrease in TSH (from 3.51 to 2.40 mU/L) after 12-weeks of Zumba training likely reflects a modulation of the HPT axis rather than a clinically significant change. Although statistically significant, this reduction should be interpreted cautiously, as it remains within normal limits (0.4‒4.0 mU/L). Nonetheless, higher TSH concentrations within the euthyroid range have been associated with less favorable cardiometabolic profiles in postmenopausal women^55^; therefore, the shift toward mid-normal values may represent improved thyroid sensitivity and metabolic regulation. Future studies assessing symptoms, metabolic rate, and quality of life are needed to determine whether such physiological adaptations have clinical relevance.

### Effect of the Zumba intervention on body composition

The present study revealed a decrease in body mass, BMI, fat mass, percentage of body fat, and waist, hip, and chest circumference following 12-weeks of Zumba training in overweight and obese postmenopausal women. The findings are consistent with previous studies of Zumba exercise training in overweight and obese women.^20^^,^^37^ In these studies, 12 to 16-weeks of Zumba training were observed to be effective in improving body composition parameters. A study by Cugusi et al.,^20^ showed significant improvements in body weight, BMI, circumferences (arm, waist, and hip), fat, and muscle mass in overweight women following a 12-week Zumba fitness program. Likewise, studies reported that an 8-week Zumba exercise program significantly reduced body mass, BMI, and fat percentage in healthy women.^22^^,^^23^ A study found that an eight-week Zumba training program had positive effects on the body composition parameters of high school students with a high body mass index, aged 15- to 17-years.^56^

It has been demonstrated that a single session of Zumba training can result in the expenditure of approximately 369 calories, with an average of 9.5 kcal per minute.^57^ Furthermore, three sessions of Zumba training per week for a period of one month can result in a loss of approximately 3 kg of body weight.^57^ Zumba is also a form of aerobic exercise,^58^ and according to Klijn et al.^59^ the rhythmic movement of large muscle groups during aerobic exercise accelerates the burning of fat, mainly subcutaneous fat, as the primary source of energy. Therefore, a Zumba training program is a highly effective intervention for improving body composition in obese women. It should be noted that the efficacy of the Zumba Fitness program is contingent on its duration. However, a study conducted over a 4- to 8-week period revealed a lack of significant effects on changes in body composition following participation in a Zumba Fitness program in adult men and women.^60^ It seems that different training intensities and durations likely caused the inconsistent results. The proposed physiological mechanisms underlying improved body composition after Zumba remain unclear. These observed beneficial effects might be explained by the impacts of exercise training on appetite control and eating behaviors in obese individuals.^61^

### Effect of the intervention on lipid profile

The present study showed that 12-week Zumba fitness training significantly reduced TG levels but had no significant effect on TC levels. Evidence on the effects of Zumba on lipids is limited. The research conducted by Araneta,^24^ demonstrated a significant decrease in triglyceride levels following a 12-week Zumba fitness intervention in sedentary obese women. A study concluded that an 8-week Zumba exercise program significantly reduced triglyceride and total cholesterol levels in sedentary women.^22^ However, after 40-weeks of Zumba training, there were no significant changes in total cholesterol and triglycerides among hospital employees.^13^ Inconsistencies between studies can be caused by various factors, such as the physical condition of the subjects, the intensity, duration, and type of exercise, age and gender differences between subjects, and even the ambient temperature. Another potential explanation is that elevated TG is associated with stress. Activities such as Zumba may indirectly facilitate their reduction by providing a source of stress relief, given that it incorporates elements of dance and rhythm, which can be a source of pleasure and a means of reducing stress.^48^ The significant interaction for TG should be interpreted in the context of substantial inter-individual variability, suggesting that factors beyond group assignment influenced the magnitude of the response.

### Limitations

The current study identified several limitations that should be considered in future research. First, although the a priori power analysis confirmed that the sample size was adequate to detect statistically significant effects for the main outcomes, the relatively small number of participants may still have limited the sensitivity to detect small effect sizes or subtle between-group differences. This constraint also increases the risk of both Type I and Type II errors despite the post-hoc power correction. Future studies should include larger and more diverse samples to confirm these findings and enhance the robustness of statistical inferences. Second, the menopausal status of participants was determined clinically rather than through biochemical verification (FSH, LH, or estradiol), which may have introduced minor misclassification, especially among women at the younger end of the age range. Third, the authors assessed body composition using skinfolds plus Siri’s equation instead of Dual-energy X-Ray Absorptiometry (DXA)/Bioelectrical Impedance Analysis (BIA), which can underestimate% fat at higher adiposity and has wider limits of agreement. To bolster reliability, the authors used a single trained assessor, calibrated calipers, and triplicate readings, but future studies should include DXA (or multi-frequency BIA with hydration control) for greater precision and validation. Fourth, the authors monitored intensity with RPE only. Although RPE correlates strongly with HR and VO_2_ and aligns with ventilatory thresholds, the absence of concurrent HR telemetry, gas exchange, or lactate sampling prevents precise quantification of the physiological stimulus. Future studies should incorporate HR (e.g.,%HR reserve), accelerometry, or submaximal gas exchange testing to verify dose fidelity. Additionally, the authors lacked a dose-controlled, modality-matched comparator; therefore, modality-specific claims are not warranted. A future randomized controlled trial should compare Zumba to another aerobic modality with matched frequency, duration, and objective intensity targets to test modality specificity. Fifth, while participants were advised to follow their habitual dietary patterns, the absence of formal dietary intake monitoring introduces a potential confounding variable, particularly in relation to fluctuations in thyroid hormone and lipid levels. Sixth, the study did not assess Low-Density Lipoprotein (LDL) cholesterol levels, a key marker of cardiovascular risk. Although TC and triglycerides TG were evaluated, the lack of LDL data limits the comprehensiveness of the lipid profile analysis. Future studies should include LDL measurements to enable a more complete assessment of cardiometabolic health outcomes in response to exercise interventions. Furthermore, the authors did not measure insulin resistance due to its close links to thyroid hormones. Finally, the deliberately homogeneous cohort (Tunisian, postmenopausal women with overweight/obesity and no major comorbidities) improves internal validity but restricts generalizability to other ethnic groups, age ranges, and individuals with common conditions (e.g., hypertension, type 2 diabetes) or relevant medications. The study was underpowered to test effect modification by baseline characteristics. Future work should be multi-center and more diverse, include participants with prevalent comorbidities and medication use, and prespecify stratified/interaction analyses and pragmatic designs to assess real-world applicability.

## Conclusions

A 12-week Zumba program was associated with favorable changes in TSH, TG levels, body composition, and blood pressure in overweight/obese postmenopausal women, with no change in FT4 or TC. These findings support aerobic dance-based group exercise delivered as Zumba as an enjoyable and accessible way to achieve a moderate-to-vigorous activity dose; however, they reflect physiological and behavioral markers from a small, short-term study and should not be interpreted as evidence of clinical treatment or prevention. Generalizability beyond the studied population remains limited, and confirmatory long-term randomized trials across broader populations (e.g., premenopausal women, men, or individuals with different health conditions) are required to establish durability, mechanisms, and clinical relevance.

## Abbreviations

ANOVA, Analysis of Variance; BMI, Body Mass Index; CG, Control Group; *d*, Cohen’s *d*; FT4, Free Thyroxine; HPT, Hypothalamic-Pituitary-Thyroid; LDL, Low-Density Lipoprotein; T3, Triiodothyronine; T4, Thyroxine; TC, Total Cholesterol; TG, Triglycerides; TRH, Thyrotropin-Releasing Hormone; TSH, Thyroid-Stimulating Hormone; RPE, Scale of Perceived Exertion; ZG, Zumba training Group; η²_p_, Partial eta-squared.

## Funding

This study was not supported by any specific grant from public, commercial, or non-profit funding entities. All resources utilized were provided by the authors' respective institutions.

Data availability statement

The datasets used and analyzed during the current study are available from the corresponding author upon reasonable request.

## Declaration of competing interest

The authors declare no conflicts of interest.

## References

[1] Greendale G.A., Huang M.H., Wight R.G. (2009). Effects of the menopause transition and hormone use on cognitive performance in midlife women. Neurology.

[2] Abildgaard J., Pedersen A.T., Green C.J. (2013). Menopause is associated with decreased whole body fat oxidation during exercise. Am J Physiol Endocrinol Metab.

[3] Monteleone P., Mascagni G., Giannini A., Genazzani A.R., Simoncini T. (2018). Symptoms of menopause ‒ global prevalence, physiology and implications. Nat Rev Endocrinol.

[4] Isacco L., Ennequin G., Boisseau N. (2023). Influence of the different hormonal status changes during their life on fat mass localisation in women: a narrative review. Arch Physiol Biochem.

[5] Britton E., Kindermann G., Domegan C., Carlin C. (2020). Blue care: a systematic review of blue space interventions for health and wellbeing. Health Promot Int.

[6] Onsori M., Galedari M. (2015). Effects of 12 weeks aerobic exercise on plasma level of TSH and thyroid hormones in sedentary women. Euro J Sports Exerc Sci.

[7] Shahid M.A., Ashraf M.A., Sharma S. (2024). http://www.ncbi.nlm.nih.gov/books/NBK500006/.

[8] Sweeney L.B., Stewart C., Gaitonde D.Y. (2014). Thyroiditis: an integrated approach. Am Fam Phys.

[9] Yang Q., Dong L.W., Yan Wan H., Yi Cao H. (2020). Association of thyroid hormones with metabolic syndrome and its components in postmenopausal Chinese women. Gynecol Endocrinol.

[10] Cooper D.S. (2004). Thyroid disease in the oldest old the exception to the rule. JAMA.

[11] Chandrashekhar G.S. (2018). Comparison of thyroid profile in premenopausal and postmenopausal women: a case control study. Int J Med Res Rev.

[12] Schindler A.E. (2003). Thyroid function and postmenopause. Gynecol Endocrinol.

[13] Barene S., Krustrup P., Jackman S.R., Brekke O.L., Holtermann A. (2014). Do soccer and Zumba exercise improve fitness and indicators of health among female hospital employees? A 12-week RCT. Scand J Med Sci Sports.

[14] Thompson W.R. (2013). Now trending: worldwide survey of fitness trends for 2014. ACSM’s Health Fitness J.

[15] Sanders M.E., Prouty J. (2012). Zumba® fitness is gold for all ages. ACSM’s Health Fitness J.

[16] Terry P.C., Karageorghis C.I., Curran M.L., Martin O.V., Parsons-Smith R.L. (2020). Effects of music in exercise and sport: a meta-analytic review. Psychol Bull.

[17] Norouzi E., Hosseini F., Vaezmosavi M., Gerber M., Pühse U., Brand S. (2020). Zumba dancing and aerobic exercise can improve working memory, motor function, and depressive symptoms in female patients with Fibromyalgia. Eur J Sport Sci.

[18] Vendramin B., Bergamin M., Gobbo S. (2016). Health benefits of Zumba fitness training: a systematic review. PMR.

[19] Bidonde J., Boden C., Busch A.J., Goes S.M., Kim S., Knight E. (2017). Dance for adults with fibromyalgia-what do we know about it? Protocol for a scoping review. JMIR Res Protoc.

[20] Cugusi L., Wilson B., Serpe R. (2016). Cardiovascular effects, body composition, quality of life and pain after a Zumba fitness program in Italian overweight women. J Sports Med Phys Fitness.

[21] Delextrat A.A., Warner S., Graham S., Neupert E. (2016). An 8-week exercise intervention based on Zumba improves aerobic fitness and psychological well-being in healthy women. J Phys Act Health.

[22] Turgut M., Soylu Y. (2021). Effects of 8-week Zumba exercise on blood lipids profile in sedentary women. Pedagogy Phys Cult Sports.

[23] Ljubojevic A., Jakovljevic V., Bijelic S. (2022). The effects of Zumba fitness® on respiratory function and body composition parameters: an eight-week intervention in healthy inactive women. Int J Environ Res Public Health.

[24] Araneta M.R., Tanori D. (2015). Benefits of Zumba Fitness® among sedentary adults with components of the metabolic syndrome: a pilot study. J Sports Med Phys Fitness.

[25] Abassi W., Ouerghi N., Ghouili H., Haouami S., Bouassida A. (2020). Greater effects of high- compared with moderate-intensity interval training on thyroid hormones in overweight/obese adolescent girls. Horm Mol Biol Clin Investig.

[26] Berahman H., Elmieh A.F., Chafy M.R. (2021). The effect of water-based rhythmic exercise training on glucose homeostasis and thyroid hormones in postmenopausal women with metabolic syndrome. Horm Mol Biol Clin Investig.

[27] Cheikh I.B., Marzouki H., Selmi O. (2025). Effect of water-based aerobic training on anthropometric, biochemical, cardiovascular, and explosive strength parameters in young overweight and obese women: a randomized controlled trial. PeerJ.

[28] Faul F., Erdfelder E., Lang A.G., Buchner A.G. (2007). *Power 3: a flexible statistical power analysis program for the social, behavioral, and biomedical sciences. Behav Res Methods.

[29] Borg G.A. (1982). Psychophysical bases of perceived exertion. Med Sci Sports Exerc.

[30] Durnin J.V., Womersley J. (1974). Body fat assessed from total body density and its estimation from skinfold thickness: measurements on 481 men and women aged from 16 to 72 years. Br J Nutr.

[31] Siri W.E. (1993). Body composition from fluid spaces and density: analysis of methods. 1961. Nutrition.

[32] Ouerghi N., Ben Fradj M.K., Talbi E., Bezrati I., Feki M., Bouassida A. (2020). Association of selected adipokines with metabolic syndrome and cardio-metabolic risk factors in young males. Cytokine.

[33] Gaskill S.E., Skinner J.S., Quindry J. (2023). Ventilatory threshold related to V.O2reserve, heart rate reserve and rating of perceived exertion in a large varied sample. Med Sci Sports Exerc.

[34] Alberton C.L., Antunes A.H., Beilke D.D. (2013). Maximal and ventilatory thresholds of oxygen uptake and rating of perceived exertion responses to water aerobic exercises. J Strength Cond Res.

[35] Scherr J., Wolfarth B., Christle J.W., Pressler A., Wagenpfeil S., Halle M. (2013). Associations between Borg’s rating of perceived exertion and physiological measures of exercise intensity. Eur J Appl Physiol.

[36] Delextrat A., Shaw C.D., Solera-Sanchez A. (2024). Heart rate responses of post-menopausal women to Zumba gold® classes. Biology.

[37] Barranco-Ruiz Y., Villa-González E. (2020). Health-related physical fitness benefits in sedentary women employees after an exercise intervention with Zumba fitness®. Int J Environ Res Public Health.

[38] Ryu H.R., Eum H.J., Kim D.Y. (2023). Assessing of exercise intensity for a rhythmik exercise program based on cardiopulmonary functions. J Exerc Rehabil.

[39] Rossmeissl A., Lenk S., Hanssen H., Donath L., Schmidt-Trucksäss A., Schäfer J. (2016). ZumBeat: evaluation of a Zumba dance intervention in postmenopausal overweight women. Sports.

[40] Cohen J. (2013).

[41] Lorestani A.Z., Rahmati M., Mirnasuri R. (2020). Effect of 12 weeks of aerobic training on liver enzymes, thyroid hormones, and anthropometric indices of obese children. Zahedan J Res Med Sci.

[42] Zenebe K., Legesse K., Mandal S., Abdulkader M., Alemu K. (2020). Impacts of resistance exercises intervention on thyroid hormone and thyroid stimulating hormone serum concentration level in school children with intellectual disabilities. Turk J Kinesiol.

[43] Onsori M., Galedari M. Effects of 12-weeks aerobic exercise on plasma level of TSH Yand thyroid hormones in sedentary women. Published online 2015.

[44] Barari A. (2016). Endurance training and ginger supplement on TSH, T3, T4 and testosterone and cortisol hormone in obese men. Persian J Med Sci.

[45] Erdogan R. (2020). Effects of endurance workouts on thyroid hormone metabolism and biochemical markers in athletes. BRAIN Broad Res Artific Intell Neurosci.

[46] Reinehr T. (2010). Obesity and thyroid function. Mol Cell Endocrinol.

[47] Babić Leko M., Gunjača I., Pleić N., Zemunik T. (2021). Environmental factors affecting thyroid-stimulating hormone and thyroid hormone levels. Int J Mol Sci.

[48] Song Q., Chen X., Su Y., Xie Z., Wang S., Cui B. (2019). Age and gender specific thyroid hormones and their relationships with body mass index in a large Chinese population. Int J Endocrinol Metab.

[49] Kiani L., Byeranvand S., Barkhordari A., Bazgir B. (2020). The effects of moderate intensity aerobic training on serum levels of thyroid hormones in inactive girls. NAEP.

[50] Luksch J.R., Collins P.B. (2018). Thyroid disorders in athletes. Curr Sports Med Rep.

[51] Soares Junior J.M., Detanac D., Sengul I., Dugalic S., Sengul D., Detanac D. (2024). Melatonin, menopause, and thyroid function in gynecologic endocrinology: what is the role?. Rev Assoc Med Bras (1992).

[52] Soares Junior J.M., Albayrak M., Sengul D., Sengul I. (2024). Thyroid function after menopause: is there any concern in thyroidology?. Rev Assoc Med Bras (1992).

[53] Sengul D., Sengul I., Soares Junior J.M (2022). Repercussion of thyroid dysfunctions in thyroidology on the reproductive system: conditio sine qua non?. Rev Assoc Med Bras.

[54] Sengul D., Sengul I. (2018). Is there any link between a kind of thyrocyte dysfunction, hypothyroidism, and inflammatory hematologic parameters in the cases possessing the benign thyroid nodules? a 5-year single-centre experience. SANAMED.

[55] Ruhla S., Weickert M.O., Arafat A.M. (2010). A high normal TSH is associated with the metabolic syndrome. Clin Endocrinol (Oxf).

[56] Kolayiş E.İ., Arol P. (2020). The effect of Zumba exercises on body composition, dynamic balance and functional fitness parameters in 15-17 years old women with high body mass index. https://acikerisim.subu.edu.tr/xmlui/handle/20.500.14002/105.

[57] Kusnanik N., Suminar T., Bird S. The effect of Zumba and high impact aerobic in reducing skinfold thickness: 2020.

[58] Sharma R., Suri M., Saini N. Physiological responses of Zumba: an overview understanding the popular fitness trend. 2017;7.

[59] Klijn P.H.C., van der Baan-Slootweg O.H., van Stel H.F. (2007). Aerobic exercise in adolescents with obesity: preliminary evaluation of a modular training program and the modified shuttle test. BMC Pediatr.

[60] Saleh O., Ljubojevic A. (2019). Effects of 12-weeks Zumba lessons on some anthropometric parameters for women and men. Assiut J Sport Sci Arts.

[61] Thivel D., Rumbold P.L., King N.A., Pereira B., Blundell J.E., Mathieu M.E. (2016). Acute post-exercise energy and macronutrient intake in lean and obese youth: a systematic review and meta-analysis. Int J Obes.

